# Systematics of a widely distributed western North American springsnail, *Pyrgulopsis micrococcus* (Caenogastropoda, Hydrobiidae), with descriptions of three new congeners

**DOI:** 10.3897/zookeys.330.5852

**Published:** 2013-09-10

**Authors:** Robert Hershler, Hsiu-Ping Liu, Corbin Bradford

**Affiliations:** 1 Department of Invertebrate Zoology, Smithsonian Institution, P.O. Box 37012, Washington, DC 20013-7012, USA; 2 Department of Biology, Metropolitan State University of Denver, Denver, CO 80217, USA

**Keywords:** *Pyrgulopsis*, Hydrobiidae, Gastropoda, United States, California, Nevada, freshwater, taxonomy, conservation

## Abstract

We describe three new species of springsnails (genus *Pyrgulopsis*) from the Amargosa River basin, California and Nevada (*P. licina* **sp. n.**, *P. perforata* **sp. n.**, *P. sanchezi* **sp. n.**), each of which was previously considered to be part of *P. micrococcus*. We also restrict *P. micrococcus* to its type locality area (Oasis Valley) and redefine a regional congener, *P. turbatrix*, to include populations from the central Death Valley region and San Bernardino Mountains that had been previously identified as *P. micrococcus*. The five species treated herein form genetically distinct lineages that differ from each other by 4.2–12.6% for mtCOI and 5.2–13.6% for mtNDI (based on previously published and newly obtained data), and are diagnosable by shell and/or penial characters. The new molecular data presented herein confirm sympatry of *P. licina* and *P. sanchezi* in Ash Meadows (consistent with morphological evidence) and delineate an additional lineage of *P. micrococcus* (in the broad sense) that we do not treat taxonomically owing to the paucity of morphological material. Conservation measures are needed to ensure the long term persistence of populations of *P. micrococcus* and a genetically differentiated lineage of *P. sanchezi* which live in disturbed habitats on private lands.

## Introduction

The western North American hydrobiid gastropod genus *Pyrgulopsis* (commonly known as springsnails) is composed of 134 currently recognized species (Hershler and Liu 2012) which typically live in springs and have very narrow geographic ranges. This large radiation is characterized by a high degree of morphological conservatism and homoplasy, which has posed difficulties in delineating species limits and phylogenetic relationships (Hershler 1994, Liu and Hershler 2005). Although the recent use of molecular tools has facilitated considerable progress in establishing a species level taxonomy that reflects the phylogenetic history of *Pyrgulopsis* (e.g., Hershler et al. 2003, Hershler and Liu 2004, Hershler and Liu 2009), a number of issues have yet to be addressed, including the unsettled status of several widely ranging congeners which have been shown to be composites of genetically divergent lineages (Hurt 2004, Liu et al. 2003, Liu and Hershler 2007). There is an urgent need to resolve these taxonomic problems to help identify conservation priorities for *Pyrgulopsis*, which is a current focus of attention of land managers owing to the increasing threats to its groundwater-dependent habitats (e.g., USFWS 2012a–c).

*Pyrgulopsis micrococcus* (Pilsbry in Stearns 1893) was originally described based on shells from two localities in the Amargosa River basin and early treated as endemic to the upper portion of this watershed (Gregg and Taylor 1965, Taylor 1985: 317). This species was subsequently revised to include additional populations scattered within large portions of the Mojave Desert (southeastern California and southwestern Nevada) that resembled specimens from the type locality area in having a globose to ovate-conic shell and distally lobate penis with a terminal gland (sometimes reduced or absent) on the ventral surface (Hershler and Sada 1987, Hershler 1989, Hershler and Pratt 1990). A recent phylogenetic analysis resolved mtDNA sequences from 29 populations of *Pyrgulopsis micrococcus* into five deeply divergent, allopatric clades–one of which also included morphologically similar and geographically proximate *Pyrgulopsis turbatrix* Hershler, 1998–which were postulated to be distinct species (Liu et al. 2003, also see Hershler and Liu 2008). In this paper we detail previously unrecognized shell and penial differences supporting recognition of three of these lineages as new species which we describe herein while also clarifying the limits of *Pyrgulopsis micrococcus* and *Pyrgulopsis turbatrix*. We also present additional molecular data that confirm sympatry of two of these novelties at various sites in Ash Meadows (consistent with our morphological evidence) and delineate a new lineage of *Pyrgulopsis micrococcus* (in the broad sense) in northern Death Valley that is not taxonomically treated owing to inadequate material.

## Methods

The previous phylogeographic investigation (Liu et al. 2003) was based on sampling across the entire broad range of *Pyrgulopsis micrococcus*, including each of the drainage basins inhabited by this species. For the current study additional molecular sampling was done to confirm the apparent sympatry of two *Pyrgulopsis micrococcus* lineages at various sites in Ash Meadows that was discovered during the course of this taxonomic study, and to evaluate the relationships of a distinctive morphotype (of *Pyrgulopsis micrococcus*) in Grapevine Springs (northern Death Valley) that was not included in our previous analysis. Several previously analyzed populations–Grapevine Springs (M2), Purgatory Spring (M8), Tecopa Spring (M25), Shoshone Spring (M26)–were also additionally sampled to increase sample size and further evaluate their genetic distinctiveness. Newly collected material was preserved in 90% ethanol in the field. Genomic DNA was extracted from entire snails (2–10 specimens per sample) using a CTAB protocol (Bucklin 1992); each specimen was analyzed for mtDNA individually. LCO1490 and HCO2198 (Folmer et al. 1994) were used to amplify a 710 base pair (bp) fragment of COI, and ND43F and RND592F (Liu et al. 2003) were used to amplify a 550 bp fragment of NADH dehydrogenase subunit I (NDI). Amplification conditions and sequencing of amplified polymerase chain reaction product followed Liu et al. (2003). Sequences were determined for both strands and then edited and aligned using Sequencherä version 5.0.1. The 51 newly sequenced specimens were analyzed together with our previously published *Pyrgulopsis micrococcus* dataset (Liu et al. 2003, Hershler and Liu 2008). The new haplotypes from each sampling locality were deposited in GenBank (accession numbers KF559184-KF559202). Sample information and GenBank accession numbers are given in Appendix I. One example of each haplotype detected in a given sample was used in our analyses.

The partition homogeneity/incongruence length difference test (Farris et al. 1994) was used to determine whether the COI and NDI datasets were consistent and could be combined for the phylogenetic analysis. The test, which was conducted using parsimony-informative sites only and 1,000 replicates, indicated no significant incongruence (*P*=0.36) and consequently we combined the two datasets in our phylogenetic analysis. MrModeltest 2.3 (Nylander 2004) was used to obtain an appropriate substitution model (using the Akaike Information Criterion) and parameter values for this analysis. Phylogenetic relationships were inferred by Bayesian analysis using MrBayes 3.1.2 (Huelsenbeck and Ronquist 2001). Metropolis-coupled Markov chain Monte Carlo simulations were run with four chains (using the model selected through MrModeltest) for 5,000,000 generations, and Markov chains were sampled at intervals of 10 generations to obtain 500,000 sample points. We used the default settings for the priors on topologies and the GTR + I + G model parameters selected by MrModeltest as the best fit model. At the end of the analysis, the average standard deviation of split frequencies was less than 0.01 (0.0063) and the Potential Scale Reduction Factor (PSRF) was 1, indicating that the runs had reached convergence. The sampled trees with branch lengths were used to generate a 50% majority rule consensus tree with the first 25% of the samples removed to ensure that the chain sampled a stationary portion. Genetic distances (maximum composite likelihood) within and between species/lineages were calculated using MEGA5 (Tamura et al. 2011), with standard errors estimated by 1,000 bootstrap replications with pairwise deletion of missing data.

Types and other voucher material were deposited in the National Museum of Natural History (USNM) collection. Relevant material from the Academy of Natural Sciences of Philadelphia (ANSP), Bell Museum of Natural History (BellMNH) and the Santa Barbara Museum of Natural History (SBMNH) was also examined during the course of this study. Series of large adults (*n*=10) were used for shell measurements. Whorl counts refer to the entire shell. Sexual dimorphism in shells, which is occasionally observed in *Pyrgulopsis* (Taylor 1987), could not be quantified owing to small sample sizes. The total number of shell whorls was counted (WH) for each specimen; and the height and width of the entire shell (SH, SW), body whorl (HBW, WBW), and aperture (AH, AW) were measured from camera lucida outline drawings using a digitizing pad linked to a personal computer (see Hershler 1989). In addition, three ratios were generated from the raw data (SW/SH, HBW/SH, AH/SH). Descriptive statistics were generated using Systat for Windows 11.00.01 (SSI 2004). Penial variation was described from series of adult specimens (typically *n*=30) that were relaxed with menthol crystals and fixed in dilute formalin prior to preservation in 70% ethanol. Descriptive penial terminology is from Taylor (1987) and Hershler (1994, 1998). Variation in the number of cusps on the radular teeth (*n*=5) was assessed using the method of Hershler et al. (2007).

We used a conservative, evolutionary lineage concept in describing new species only for those snails that are morphologically diagnosable as well as phylogenetically independent and substantially divergent genetically (Hershler et al. 2007). Inasmuch as the principal goal of our paper is to delimit species, we provide only brief taxonomic descriptions which focus on those aspects of morphology that have proven most useful in previous such studies of *Pyrgulopsis* (Taylor 1987, Hershler 1994, Hershler 1998).

## Results

**Figure 1.**
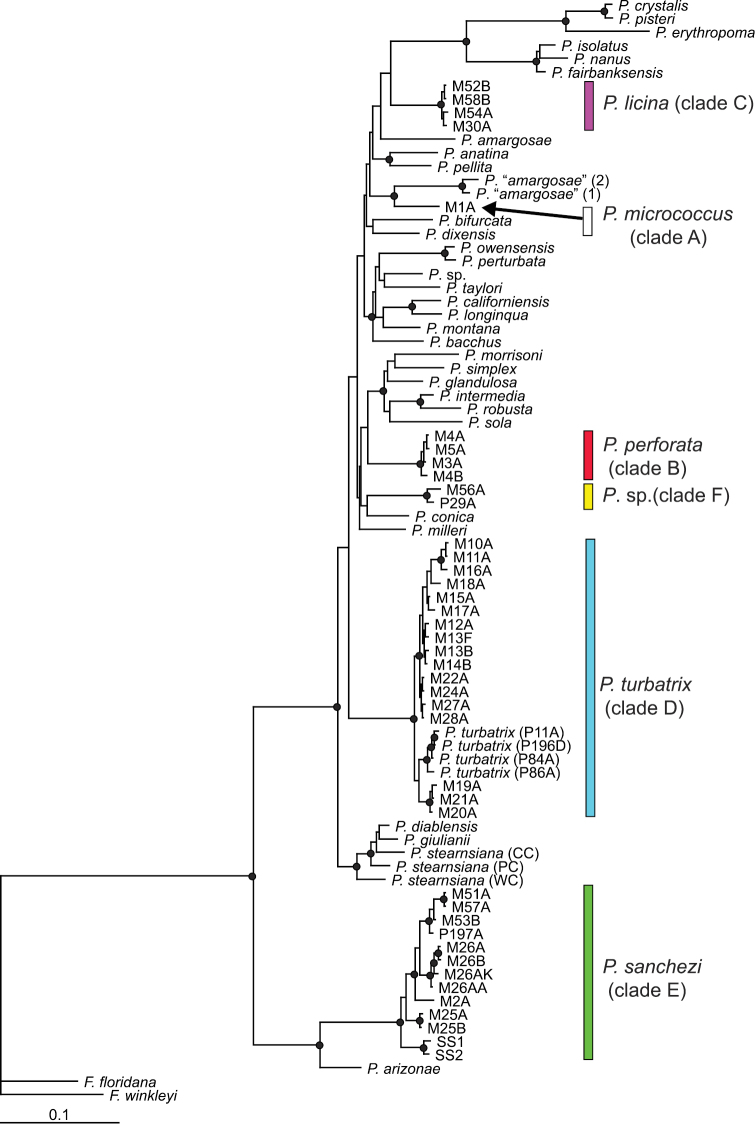
Bayesian tree based on the combined (COI, NDI) dataset. Nodes having posterior probabilities >95% are identified by filled circles. Specimen codes are from Appendix I.

The alignment of COI and NDI sequences yielded 1188 bp. The five previously reported clades (A–E) were similarly recovered in the Bayesian analysis of this combined dataset (Fig. 1). The *Pyrgulopsis micrococcus* morphotype in Grapevine Springs which was not included in our prior analysis formed an additional lineage (F) together with specimens from a spring in the Southern California coastal drainage. This clade is not formally treated herein owing to the paucity of morphological material. The additional molecular sampling conducted for this study also confirmed sympatry of morphologically distinctive clades C and E at three localities in Ash Meadows (M51–52, M53–54, M57–58) (Fig. 1), providing additional support for recognizing these as separate species.

**Figure 2.**
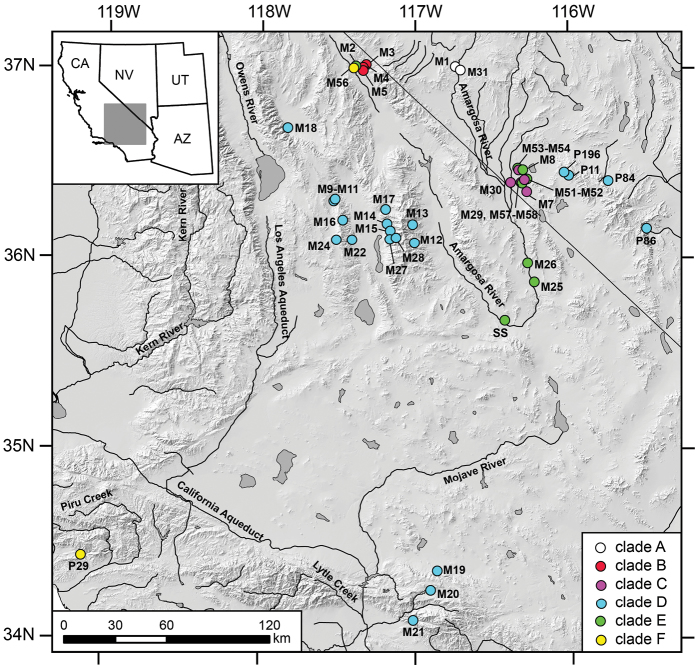
Map showing the distribution of mtDNA clades **A–F** with color codes matching those in Fig. 1.

Clades A–F differed from each other by 4.2–12.6% for COI and 5.2–13.6% for NDI; variation within clades ranged from 0–2.5% for COI and 0–3.5% for NDI (Appendix II). The geographic distributions of these genetic lineages are shown in Fig. 2. Based on the genetic evidence of distinctiveness and diagnosable shell and/or penial characters (detailed below) we recognize three of these lineages as new species which are described below (clade B as *Pyrgulopsis perforata*, clade C as *Pyrgulopsis licina*, clade E as *Pyrgulopsis sanchezi*), restrict *Pyrgulopsis micrococcus* to its type locality area (Oasis Valley, clade A), and revise *Pyrgulopsis turbatrix* to include populations from the central Death Valley region and San Bernardino Mountains that had been previously identified as *Pyrgulopsis micrococcus* (clade D).

## Systematic descriptions

### Family Hydrobiidae
Subfamily Nymphophilinae
Genus *Pyrgulopsis* Call & Pilsbry, 1886

#### Pyrgulopsis licina

Hershler, Liu & Bradford sp. n.

http://zoobank.org/5F006641-38E0-4AA0-B3D2-AE78145B51CC

http://species-id.net/wiki/Pyrgulopsis_licina

Figs 3 , 4A–B

Pyrgulopsis micrococcus . – Hershler and Sada 1987 (in part).
Pyrgulopsis micrococcus clade C. – Liu et al. (2003).

##### Types.

Holotype, USNM 850347 (a dry shell), spring south of Clay Pits, Ash Meadows, Nye County, Nevada, 36.40719°N, 116.37856°W, 11 November 1985, R. Hershler and D. W. Sada. Paratypes, USNM 1204732 (from same lot).

##### Referred material.

NEVADA. *Nye County*: USNM 859186, USNM 903997, spring south of Clay Pits, USNM 850345, USNM 850346, USNM 850347, USNM 850348, USNM 859185, spring at Clay Pits, Ash Meadows (36.41608°N, 116.37802°W), USNM 850343, USNM 850344, USNM 859184, spring north of Clay Pits, Ash Meadows (36.41613°N, 116.37808°W), USNM 850334, Rogers Spring, Ash Meadows (36.47931°N, 116.32622°W), USNM 850336, USNM 1122742, USNM 1122754, USNM 1197782, USNM 1204745, springs south of Rogers Spring, Ash Meadows (36.47467°N, 116.32747°W), USNM 850349, USNM 1197775, spring east of Crystal Reservoir, Ash Meadows (36.40790°N, 116.31297°W), USNM 850350, spring east of Crystal Reservoir, Ash Meadows (36.40742°N, 116.31197°W), USNM 903982, spring east of Crystal Reservoir, Ash Meadows (36.40836°N, 116.31042°W), USNM 1197780, spring ca. 100 m north of Collins Ranch, Ash Meadows (36.42038°N, 116.29921°W), USNM 850352, USNM 850351, USNM 859188, USNM 859189, USNM 1122848, Frenchy Springs, Ash Meadows (36.36364°N, 116.27432°W), USNM 850353, USNM 859190, USNM 894336, USNM 1122849, Last Chance Spring, Ash Meadows (36.35700°N, 116.27400°W).

##### Diagnosis.

A small congener (maximum shell height, 2.4 mm) having a narrow-conic shell. Distinguished from similar regional species by its strongly curved penial filament and absence of glands on the penis. Further differentiated from frequently sympatric *Pyrgulopsis sanchezi* (described below) by its highly convex, deeply incised teleoconch whorls and ovate shell aperture.

##### Description.

**Figure 3.**
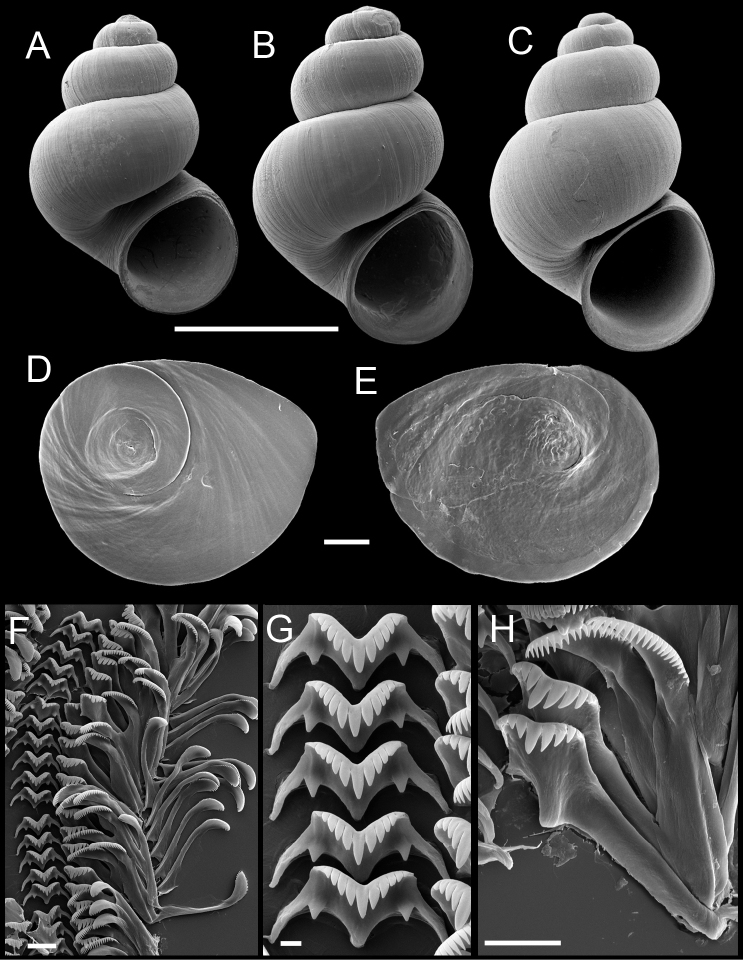
Shells, opercula and radula, *Pyrgulopsis licina* sp. n. **A** Holotype, USNM 850347 **B, C** Shells, USNM 1204732, USNM 1188732 **D, E** Opercula (outer, inner sides), USNM 850348 **F** Portion of radular ribbon, USNM 850348 **G** Central teeth, USNM 850348 **H** Lateral and inner marginal teeth, USNM 850348. Scale bars **A–C** 1.0 mm; **D, E** 100 µm; **F, H** 10 µm; **G** 2 µm.

Shell (Fig. 3A–C) narrow-conic, whorls 3.75–4.50. Teleoconch whorls highly convex, sutures deeply impressed. Aperture ovate, parietal lip complete, narrowly adnate or slightly disjunct, umbilicus narrow. Outer lip thin, orthocline or prosocline. Sculpture of faint, irregular spiral striae.

Operculum (Fig. 3D–E) as for genus; edges of last 0.5 whorl frilled on outer side; muscle attachment margins variably thickened on inner side. Radula (Fig. 3F–H) as for genus; dorsal edge of central teeth concave, lateral cusps four–six, basal cusp one. Lateral teeth having three–four cusps on both inner and outer sides. Inner marginal teeth with 20–25 cusps, outer marginal teeth with 24–31 cusps. Radula data are from USNM 850348.

**Figure 4.**
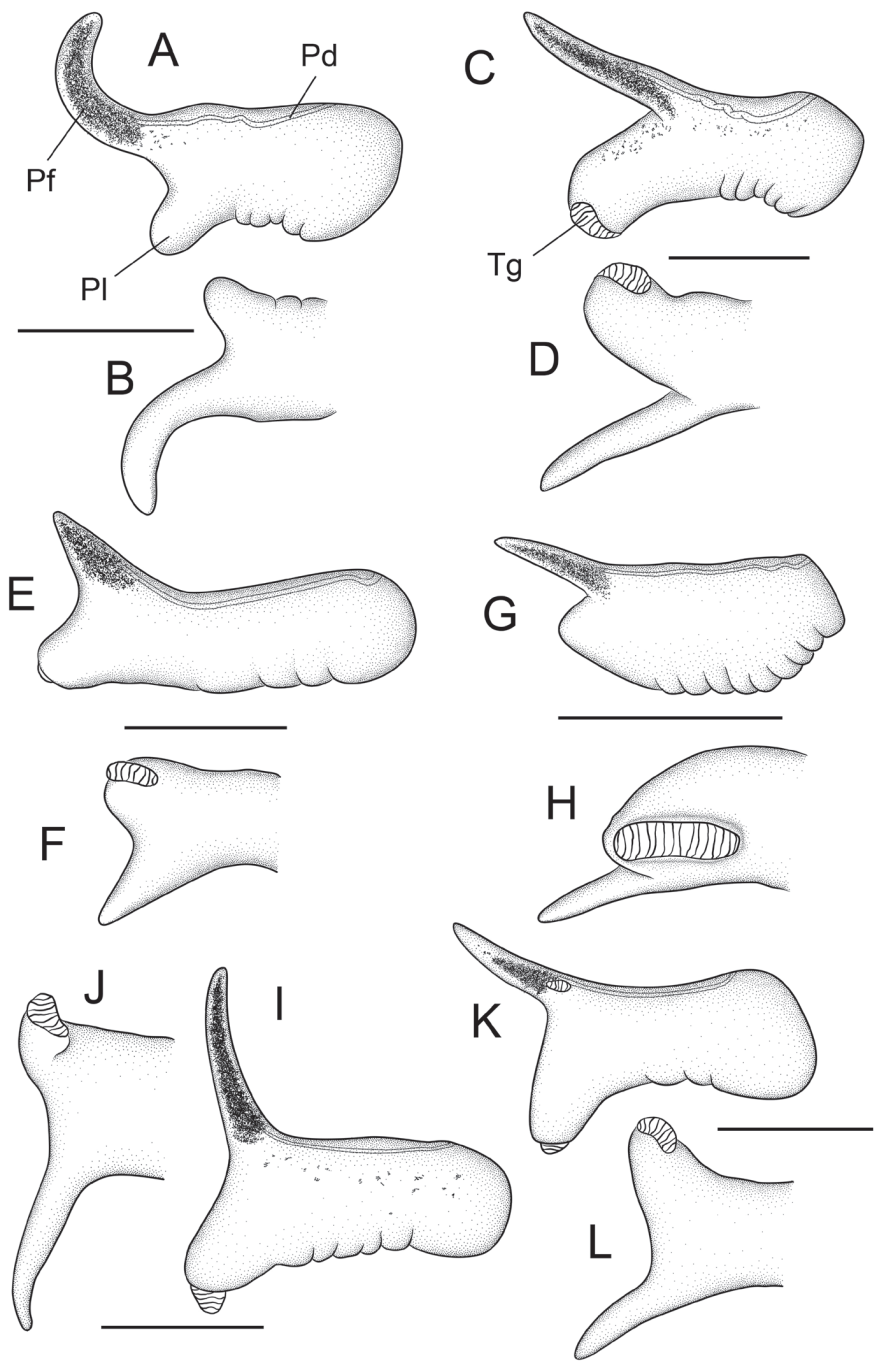
Penes (dorsal, ventral surfaces). **A, B** *Pyrgulopsis licina* sp. n., USNM 850346 **C, D** *Pyrgulopsis perforata* sp. n., BellMNH 20891 **E, F** *Pyrgulopsis sanchezi* sp. n., USNM 883361 **G, H** *Pyrgulopsis micrococcus*, BellMNH 20663 **I, J** *Pyrgulopsis turbatrix*, USNM 860699 **K, L** *Pyrgulopsis turbatrix*, USNM 883373. Scale bars **A–C** 250 µm; **D–L** 500 µm. **Pd** penial duct **Pf** penial filament **Pl** penial lobe **Tg** terminal gland.

Penis (Fig. 4A–B) medium-sized; filament medium length, narrow, weakly tapering, strongly curved (to outer side); lobe small, rectangular, horizontal or oblique; glands almost always absent (87/90 specimens), two specimens had a small, dot-like gland along the distal edge of the lobe and one specimen had a glandular smear near the distal edge of the ventral surface of the lobe. Penial data are from USNM 850334, USNM 850348, USNM 850351.

**Table 1.** Shell parameters for *Pyrgulopsis licina*. Measurements are in mm.<br/>

	WH	SH	SW	HBW	WBW	AH	AW	SW/SH	HBW/SH	AH/SH
Holotype, USNM 850347										
	4.25	1.94	1.33	1.42	1.09	0.78	0.76	0.69	0.73	0.44
USNM 1204732 (n=10)										
Mean	4.00	2.00	1.33	1.45	1.15	0.83	0.76	0.67	0.73	0.42
S.D.	0.12	0.12	0.06	0.08	0.06	0.05	0.03	0.03	0.02	0.02
Range	3.75–4.25	1.88–2.22	1.28–1.44	1.34–1.60	1.09–1.27	0.75–0.92	0.72–0.83	0.63–0.72	0.70–0.76	0.38–0.45

##### Etymology.

The epithet is an adjective derived from the New Latin *licinus*, meaning bent or turned upward, and refers to the distinctive shape of the penial filament in this species.

##### Distribution.

**Figure 5.**
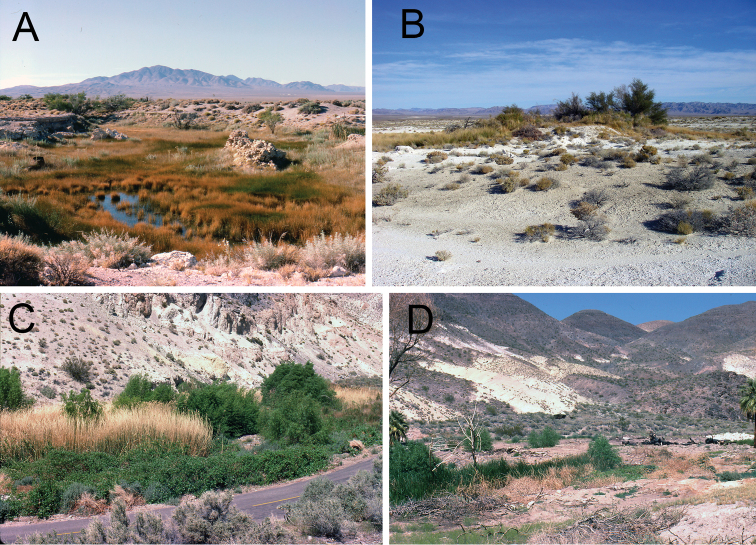
Photographs of habitats. **A** Spring south of Clay Pits, Ash Meadows, Nye County, Nevada, type locality of *Pyrgulopsis licina* sp. n. (photograph taken on 7/VII/1986) **B** Purgatory Spring, Ash Meadows, Nye County, Nevada, type locality of *Pyrgulopsis sanchezi* (15/XI/2011) **C, D** Uppermost spring east of Scotty’s Castle, Death Valley, Inyo County, California, type locality of *Pyrgulopsis perforata* sp. n. (18/IV/1980).

Ash Meadows, Amargosa River basin (M7, M29, M30, M52, M54, M58, Fig. 2). The type locality is a broad spring brook that courses through a pit-like depression (Fig. 5A).

##### Remarks.

The relationships of *Pyrgulopsis licina* were not well resolved in the molecular phylogenetic analysis (Fig. 1). Haplotype variation within this clade was relatively small (Appendix II).

#### Pyrgulopsis perforata

Hershler, Liu & Bradford sp. n.

http://zoobank.org/B246F89B-53AD-4476-8F47-DE21324ED20F

http://species-id.net/wiki/Pyrgulopsis_perforata

Figs 4C–D , 6

Pyrgulopsis micrococcus . – Hershler 1989 (in part).
Pyrgulopsis micrococcus clade B. – Liu et al. (2003).

##### Types.

**United States**: Holotype, USNM 853507 (a dry shell), easternmost spring from Scotty’s Castle along California Highway 72, Grapevine Canyon, Death Valley, Inyo County, California, 37.03233°N, 117.32333°W, 26 February 1985, R. Hershler. Paratypes, USNM 1204734 (from same lot).

##### Referred material.

CALIFORNIA. *Inyo County*: BellMNH 20891, USNM 857965, USNM 883371, USNM 883374, USNM 883375, USNM 883376, USNM 883377, USNM 894332, easternmost spring from Scotty’s Castle along CA Hwy 72, Grapevine Canyon, Death Valley, USNM 883369, spring east of Scotty’s Castle along CA Hwy 27, Grapevine Canyon, Death Valley (37.03259°N, 117.33118°W), USNM 883368, USNM 883379, spring just east of Scotty’s Castle, Grapevine Canyon, Death Valley (37.03205°N, 117.33715°W), BellMNH 20999, USNM 894333, spring ca. 0.8 km west of Scotty’s Castle along CA Hwy 72, Grapevine Canyon, Death Valley (37.01400°N, 117.34867°W), USNM 894334, Surprise Springs, Death Valley (36.99933°N, 117.34400°W).

##### Diagnosis.

A small to medium-sized congener (maximum shell height, 2.6 mm) having a broadly to ovate conic shell. Differentiated from similar regional species except *Pyrgulopsis micrococcus* by its low-spired, broadly umbilicate shell. Differs from *Pyrgulopsis micrococcus* in having a larger distal lobe and smaller gland on the penis.

##### Description.

**Figure 6.**
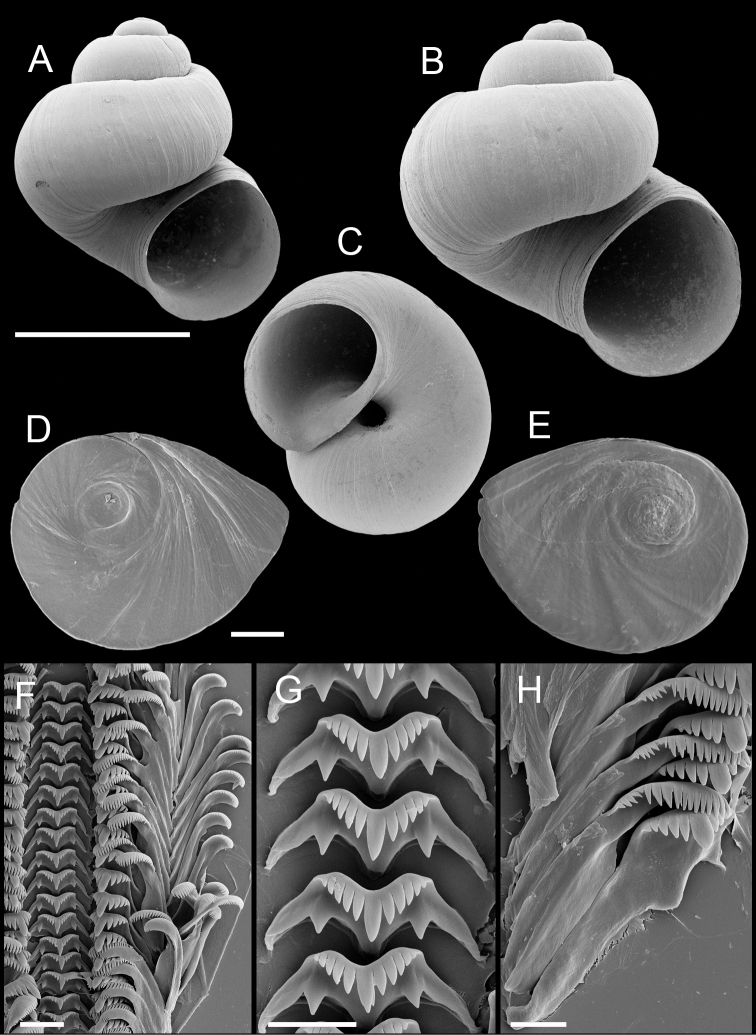
Shells, opercula and radula, *Pyrgulopsis perforata* sp. n. **A** Holotype, USNM 853507 **B, C** Shells, USNM 1204734 **D, E** Opercula (outer, inner sides), USNM 857965 **F** Portion of radular ribbon, USNM 857965 **G** Central teeth, USNM 857965 **H** Lateral and inner marginal teeth, USNM 857965. Scale bars **A–C** 1.0 mm; **D, E** 200 µm; **F** 20 µm; **G, H** 10 µm.

Shell (Fig. 6A–B) broadly to ovate conic, whorls 3.00–4.25. Teleoconch whorls medium convex, shouldered. Aperture ovate, parietal lip complete, narrowly adnate or slightly disjunct, last 0.25–0.5 whorl rarely loosened behind aperture, umbilicus broad (Fig. 6C). Outer lip thin, orthocline or prosocline.

Operculum (Fig. 6D–E) as for genus; outer side smooth; inner side smooth or weakly thickened along portions of the muscle attachment margin. Radula (Fig. 6F–H) as for genus; dorsal edge concave, lateral cusps four–eight, basal cusp one. Lateral teeth having two–four cusps on inner sides and three–six cusps on outer sides. Inner marginal teeth with 14–24 cusps, outer marginal teeth with 18–31 cusps. Radula data are from USNM 857965.

Penis (Fig. 4C–D) medium-sized; filament medium length, narrow, tapering, oblique; lobe medium-sized, rectangular, horizontal or slightly oblique; small (terminal) gland present on ventral edge of lobe (60/60 specimens), one specimen had an additional dot-like gland on the ventral surface of the lobe and one specimen had a similar glandular unit on the dorsal surface of the lobe. Penial data are from BellMNH 20891, USNM 883371.

**Table 2.** Shell parameters for *Pyrgulopsis perforata*. Measurements are in mm.<br/>

	WH	SH	SW	HBW	WBW	AH	AW	SW/SH	HBW/SH	AH/SH
Holotype, USNM 853507										
	3.75	1.82	1.51	1.46	1.30	0.87	0.79	0.83	0.80	0.48
USNM 1204734 (n=10)										
Mean	3.63	1.83	1.52	1.50	1.29	0.91	0.82	0.84	0.82	0.50
S.D.	0.21	0.21	0.08	0.13	0.09	0.07	0.05	0.08	0.03	0.04
Range	3.25–4.00	1.51–2.09	1.41–1.67	1.31–1.68	1.15–1.41	0.83–1.03	0.75–0.90	0.74–0.98	0.77–0.87	0.45–0.55

##### Distribution.

Lower portion of Grapevine Canyon, and Grapevine Mountains, lower Amargosa River basin (M3, M4, M5, Fig. 2). The type locality (Fig. 5C–D) is the uppermost of a small series of springs to the east of Scotty’s Castle.

##### Etymology.

An adjective derived from the New Latin, *perforare*, meaning to pierce, and referring to the broad umbilicate shells of this species.

##### Remarks.

The relationships of *Pyrgulopsis perforata* were not well resolved in the molecular phylogenetic analysis (Fig. 1). Haplotype variation within this clade was relatively small (Appendix II).

#### Pyrgulopsis sanchezi

Hershler, Liu & Bradford sp. n.

http://zoobank.org/DA1A41D8-E257-466B-B28E-E500E89D16A9

http://species-id.net/wiki/Pyrgulopsis_sanchezi

Figs 4E–F , 7

Pyrgulopsis micrococcus . – Hershler and Sada 1987 (in part).
Pyrgulopsis micrococcus . – Hershler 1989 (in part).
Pyrgulopsis micrococcus clade E. – Liu et al. (2003).

##### Types.

**United States**: Holotype, USNM 850333 (a dry shell), Purgatory Spring, Ash Meadows, Nye County, Nevada, 36.47200°N, 116.31617°W, 26 February 1985, R. Hershler and D.W. Sada. Paratypes, USNM 1204735 (from same lot).

##### Referred material.

CALIFORNIA. *Inyo County*: USNM 853505, USNM 853506, USNM 854609, USNM 854610, Grapevine Springs, spring brook on travertine bench above Scotty’s Ranch, Death Valley (37.019210°N, 117.38649°W), USNM 857964, USNM 883372, USNM 1152507, Grapevine Springs, spring outflow at Scotty’s Ranch, Death Valley (37.01830°N, 117.38770°W), USNM 1197772, Grapevine Springs, spring outflow below Scotty’s Ranch, Death Valley (37.01760°N, 117.39420°W), USNM 853503, Grapevine Springs, northern-most spring complex, outflow below base of hill, Death Valley (37.01970°N, 117.39288°W), USNM 894331, Grapevine Springs, third stream north of ranch, Death Valley (37.01867°N, 117.38900°W), BellMNH 21116, USNM 853501, USNM 857962, USNM 894335, USNM 1152506, Shoshone Spring, (35.98022°N, 116.27308°W), USNM 853502, USNM 857963, USNM 873153, USNM 883366, USNM 894354, Tecopa Hot Springs, northern-most spring, (35.88011°N, 116.22992°W), USNM 874035, Spring brook north of Tecopa, (35.85346°N, 116.22361°W). *San Bernardino County*: USNM 123904, USNM 883365, USNM 899902, USNM 1008345, USNM 1008725, USNM 1011485, USNM 1152503, Saratoga Springs, Death Valley (35.68099°N, 116.42245°W). NEVADA. *Nye County*: USNM 850339, USNM 859183, USNM 1122825, Shaft Spring, Ash Meadows (36.45109°N, 116.31552°W), USNM 850340, USNM 1122826, Chalk Spring, Ash Meadows (36.44913°N, 116.31497°W), BellMNH 20664, School Spring, Ash Meadows (36.42741°N, 116.30397°W), USNM 1204746, Rogers Spring, Ash Meadows (36.47931°N, 116.32632°W), USNM 850335, USNM 859180, USNM 859181, USNM 204755, USNM 1122554, springs south of Rogers Spring, Ash Meadows (36.47467°N, 116.32747°W), USNM 850337, USNM 850338, Five Springs, Ash Meadows (36.46476°N, 116.32023°W), USNM 859182, USNM 1122821, spring south of Five Springs, Ash Meadows (36.45109°N, 116.31552°W), BellMNH 20666, BellMNH 20743, BellMNH 21149, USNM 850341, USNM 850342, USNM 859195, USNM 1204752, spring ca. 100 m north of Collins Ranch, Ash Meadows (36.42038°N, 116.29921°W), BellMNH 20741, USNM 859179, USNM 883361, USNM 894337, USNM 1074313, USNM 1122759, USNM 1152498, Purgatory Spring, Ash Meadows, USNM 1204738, USNM 1197773, spring east of Crystal Reservoir, Ash Meadows (36.40790°N, 116.31297°W), USNM 859187, USNM 1204744, spring east of Crystal Reservoir, Ash Meadows (36.40742°N, 116.31197°W).

##### Diagnosis.

A small to medium-sized congener (maximum shell height, 2.9 mm) having an ovate to narrow conic shell. Differentiated from similar regional species by its short, strongly tapering penial filament.

##### Description.

**Figure 7.**
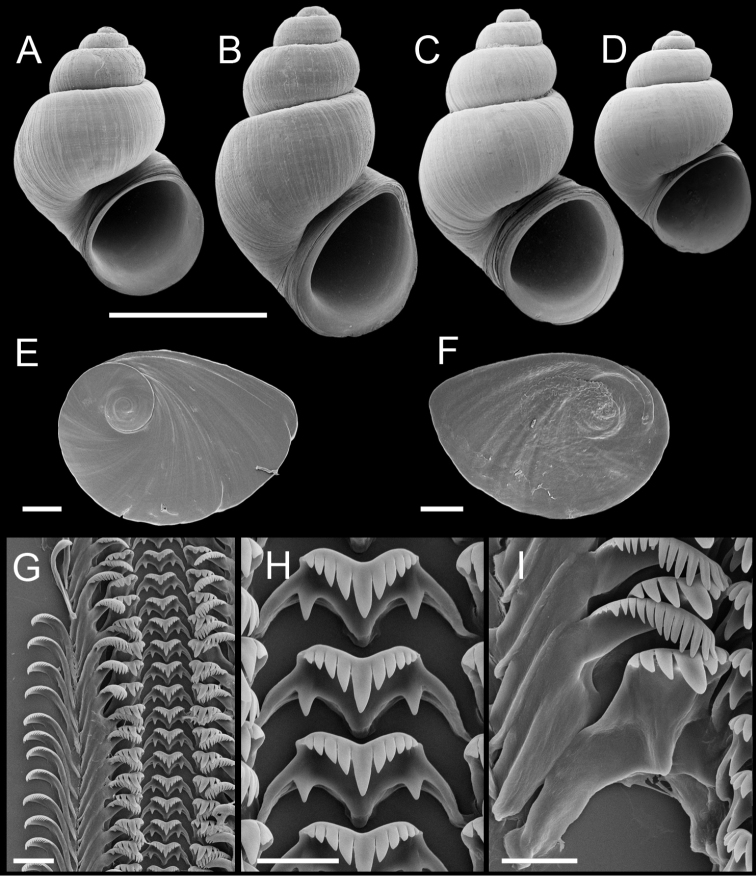
Shells, opercula and radula, *Pyrgulopsis sanchezi* sp. n. **A** Holotype, USNM 850333 **B–D** Shells, USNM 853505, USNM 1204755, USNM 853501 **E, F** Opercula (outer, inner sides), USNM 883361 **G** Portion of radular ribbon, USNM 883361 **H** Central teeth, USNM 883361 **I** Lateral and inner marginal teeth, USNM 883361. Scale bars **A–D** 1.0 mm; **E, F** 100 µm; **G** 20 µm; **H, I** 10 µm.

Shell (Fig. 7A–D) ovate to narrow conic, whorls 3.5–4.75. Teleoconch whorls medium convex, sometimes strongly shouldered, last 0.25–0.50 whorl sometimes slightly loosened. Aperture ovate, sometime strongly angled adapically, parietal lip complete, narrowly adnate or slightly disjunct, umbilicus usually narrow. Apertural lip sometimes rather thickened and/or slightly reflected, outer lip orthocline or prosocline.

Operculum (Fig. 7E–F) as for genus; outer side smooth or with last 0.5 whorl weakly frilled; inner side smooth or slightly thickened along a small portion of the muscle attachment margin. Radula (Fig. 7G–I) as for genus; dorsal edge concave, lateral cusps three–six, basal cusp one. Lateral teeth having one–four cusps on inner sides and two–six cusps on outer sides. Inner marginal teeth with 10–26 cusps, outer marginal teeth with 12–33 cusps. Radula data are from BellMNH 21116, USNM 857963, USNM 883361, USNM 883365, USNM 883372.

Penis (Fig. 4E–F) medium-sized; filament short, broad, strongly tapering, oblique; lobe short, rectangular, horizontal or slightly oblique; small (terminal) ovate gland almost always present on ventral surface of lobe (92/93 specimens), gland usually positioned horizontally, rarely borne on a raised swelling (one specimen), one specimen had a second, dot-like gland on the ventral surface of the lobe. Penial data are from BellMNH 2116, USNM 857963, USNM 857964, USNM 883361, USNM 883666.

**Table 3.** Shell parameters for *Pyrgulopsis sanchezi*. Measurements are in mm. <br/>

	WH	SH	SW	HBW	WBW	AH	AW	SW/SH	HBW/SH	AH/SH
Holotype, USNM 850333										
	4.25	2.50	1.73	1.95	1.40	1.10	1.07	0.69	0.78	0.44
USNM 1204735 (n=10)										
Mean	4.33	2.43	1.66	1.82	1.33	1.06	0.99	0.68	0.75	0.43
S.D.	0.21	0.14	0.10	0.10	0.06	0.06	0.07	0.04	0.02	0.02
Range	4.00–4.75	2.22–2.70	1.46–1.81	1.68–1.94	1.22–1.44	0.96–1.16	0.90–1.08	0.62–0.73	0.70–0.78	0.40–0.46

##### Distribution.

Distributed in five separate groundwater discharge areas of the Amargosa River basin: Grapevine Springs (M2), Ash Meadows (M8, M51, M53, M57), Tecopa (M25), Shoshone (M26), Saratoga Spring (SS) (Fig. 2). The type locality (Fig. 5B) is a flowing well that was drilled into a small spring mound (Dudley and Larson 1976).

##### Etymology.

This species is named for Peter G. Sanchez, who spearheaded early efforts to protect and conserve regional springsnails and their associated aquatic habitats while serving as a Resource Management Specialist in the Death Valley National Monument (now National Park) and Chair of the Desert Fishes Council (1978–1980).

##### Remarks.

*Pyrgulopsis sanchezi* was resolved as sister to *Pyrgulopsis arizonae* (Gila River basin, Arizona) in the Bayesian analysis (Fig. 1). The five geographically separated groups of *Pyrgulopsis sanchezi* populations are genetically differentiated—e.g., mean genetic distance is 1.5+/-0.3% (ranging from 1.3–2.3%) for COI and 2.1+/-0.6% (ranging from 1.8–3.2%) for NDI, however we have not found consistent morphological differences to support their recognition as distinct species.

#### Pyrgulopsis micrococcus

(Pilsbry, 1893)

http://species-id.net/wiki/Pyrgulopsis_micrococcus

Figs 4G–H , 8

Amnicola micrococcus Pilsbry in Stearns 1893: 277, fig. 1 (small spring in Oasis Valley Nevada; also from Death Valley). Baker 1964: 174 (lectotype designation).
Fontelicella (Microamnicola) micrococcus . – Gregg and Taylor 1965: 109 (comb. n.).
Pyrgulopsis micrococcus . – Hershler and Thompson 1987: 29 (new combination). Hershler and Sada 1987: 788–791 (in part). Hershler 1989: 182–187 (in part). Hershler 1998: 15.
Pyrgulopsis micrococcus clade A. – Liu et al. (2003).

##### Types.

Lectotype, ANSP 67279; paralectotypes, ANSP 368399, USNM 123622 (from same lot).

##### Other material examined.

NEVADA. *Nye County*: USNM 847246, springs at Springdale (37.03049°N, 116.75117°W), BellMNH 20674, spring east of Springdale (37.03858°N, 116.71730°W), BellMNH 20671, BellMNH 20672, BellMNH 20673, BellMNH 20739, USNM 850297, USNM 857961, USNM 874778, USNM 894330, USNM 905091, USNM 1002348, USNM 1004184, USNM 1004185, USNM 1068649, USNM 1068650, USNM 1068794, spring at Fleur de Lis Ranch, ca. 0.8 km south of Springdale (37.01700°N, 116.73300°W), BellMNH 20670, USNM 874771, USNM 1002349, USNM 1004182, USNM 1004183, USNM 1146338, Goss Springs (36.99906°N, 116.70725°W), BellMNH 20669, BellMNH 20774, Ute Springs (36.95729°N, 116.71648°W), BellMNH 20667, BellMNH 20740, USNM 874758, Revert Springs (36.91795°N, 116.74397°W).

##### Revised diagnosis.

A medium-sized congener (maximum shell height, 4.4 mm) having a broadly to elongate conic shell. Differentiated from similar regional species by the large size of the gland on the ventral surface of the penis.

##### Description.

**Figure 8.**
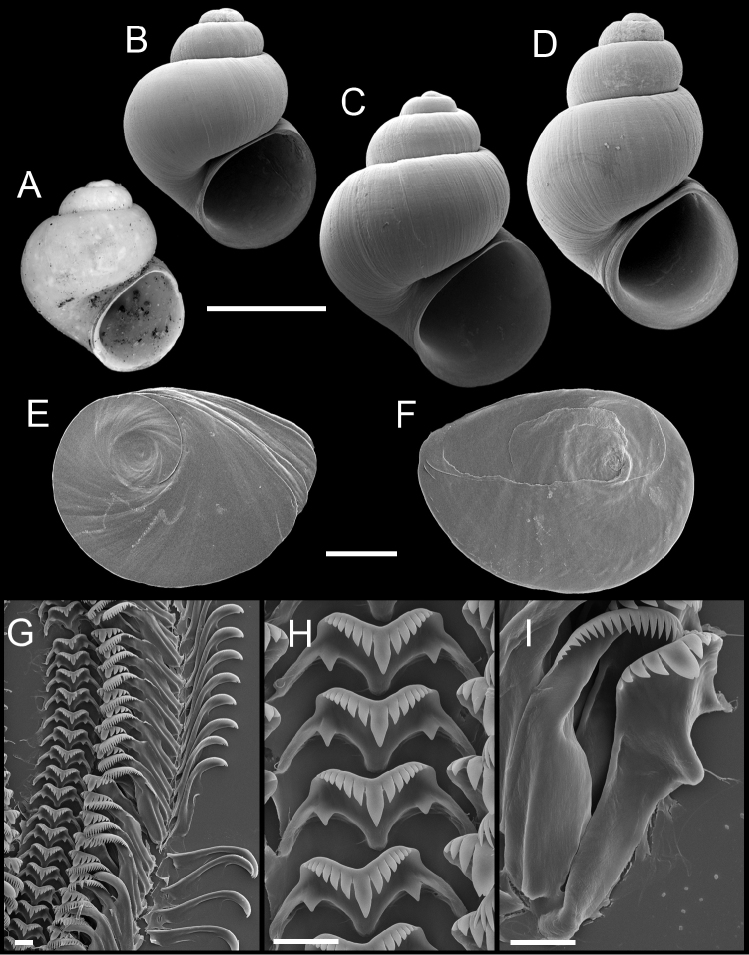
Shells, opercula and radula, *Pyrgulopsis micrococcus*. **A** Lectotype, ANSP 67279a **B–D** Shells, USNM 1004185, USNM 905091, USNM 1004183 **E, F** Opercula (outer, inner sides), USNM 847246 **G** Portion of radular ribbon, USNM 847246 **H** Central teeth, USNM 847246 **I** Lateral and inner marginal teeth, USNM 847246. Scale bars **A–D** 1.0 mm; **E, F** 250 µm; **G–I** 10 µm.

Shell (Fig. 8A–D) broadly to narrow-conic, whorls 3.50–5.0. Teleoconch whorls weakly to strongly convex, sutures impressed. Aperture ovate, parietal lip complete, usually disjunct, last 0.25–0.5 whorl often loosened behind aperture, umbilicus small. Outer lip usually thin, orthocline. Sculpture of faint, irregular spiral striae.

Operculum (Fig. 8E–F) as for genus; edges of last 0.5 whorl frilled on outer side; muscle attachment margins thickened on inner side. Radula (Fig. 8G–I) as for genus; dorsal edge of central radular teeth concave, lateral cusps five–eight, basal cusp one. Lateral teeth having three–four cusps on inner sides and four–five cusps on outer sides. Inner marginal teeth with 18–23 cusps, outer marginal teeth with 21–29 cusps. Radula data are from USNM 847246.

Penis (Fig. 4G–H) medium-sized; filament short, narrow, tapering, slightly oblique; lobe small, tapering, horizontal; a large (terminal) gland (borne on a raised swelling) present on ventral surface of penis, extending from near mid-length almost to tip of lobe (90/90 specimens), one–two additional small glands sometimes present on ventral surface of lobe (8 specimens), one specimen had a glandular dot on the dorsal surface near the base of the filament. Penial data are from BellMNH 20663, BellMNH 20669, BellMNH 20744.

##### Distribution.

Several groups of springs in Oasis Valley, upper Amargosa River basin (M1, M31, Fig. 2).

##### Remarks.

Pilsbry (in Stearns 1893; also see Stearns 1901) listed a single “type” lot for *Pyrgulopsis micrococcus*, [USNM] 123622, which is composed of six dry shells. Baker (1964) subsequently designated ANSP 67279a as the “type” without explaining his rationale for this action. ANSP 67279a (Fig. 8A) closely conforms to Pilsbry’s description and figure and is also very similar to the USNM type material (Hershler and Sada 1987, fig. 8a). The labels associated with ANSP 67279a indicate that it was part of the original collection of *Pyrgulopsis micrococcus* (made by C. Hart Merriam) and this lot was almost certainly known to Pilsbry, who was the curator of mollusks at the Academy of Natural Sciences during the time period when his description was prepared and published. Based on this evidence we conclude that ANSP 67279a is part of the type series and thus Baker’s subsequent lectotype designation is valid.

*Pyrgulopsis micrococcus* was resolved in the Bayesian tree as sister to an undescribed species from the Amargosa Canyon, south of Tecopa (Fig. 1). Specimens assigned to *Pyrgulopsis micrococcus* vary somewhat in size and shell shape, but are closely similar both genetically (Liu et al. 2003) and in penial morphology.

#### Pyrgulopsis turbatrix

Hershler, 1998

http://species-id.net/wiki/Pyrgulopsis_turbatrix

Figs 4I–L , 9

Pyrgulopsis turbatrix Hershler, 1998: 50, figs. 6K, 18G–J, 30D–F (Horseshutem Springs, Pahrump Valley, Nye County, Nevada).
Pyrgulopsis micrococcus . – Hershler 1989 (in part).
Pyrgulopsis micrococcus . – Hershler and Pratt 1990 (in part).
Pyrgulopsis micrococcus clade D. – Liu et al. (2003).

##### Types.

Holotype, USNM 883978; paratypes, USNM 860699 (from same lot).

##### Other material examined.

CALIFORNIA. *Inyo County*: USNM 853508, USNM 883373, Hanaupah Spring, Hanaupah Canyon, Death Valley (36.18684°N, 117.02537°W), USNM 853512, spring above Darwin Falls, Panamint Valley (36.31783°N, 115.52700°W), USNM 857969, stream below Darwin Falls, Panamint Valley (36.32033°N, 117.51917°W), USNM 857968, Saline Marsh, Saline Valley (36.69350°N, 117.83033°W). *San Bernardino County*: SBMNH uncat., roadside spring between north shore highway and Big Bear Lake at point 1.2 km east of road which crosses lake, Southern California coastal drainage (34.26424°N, 116.87497°W), USNM 860450, spring southwest of Big Bear Ranger Station, southern California coastal drainage (34.26281°N, 116.90185°W). NEVADA. *Clark County*: USNM 883551, Willow Spring, Indian Springs Valley (36.41700°N, 115.76419°W), USNM 883981, La Madre Spring, Las Vegas Valley (36.18381°N, 115.50638°W), USNM 1002785, Harris Spring, Las Vegas Valley (36.24071°N, 115.54351°W). *Nye County*: USNM 854967, Wood Canyon Spring, Pahrump Valley (36.39924°N, 115.93258°W).

##### Revised diagnosis.

A medium-sized congener (maximum shell height, 3.7 mm) having an ovate to narrow-conic shell. Differentiated from similar regional species by the combination of its relatively large, narrow shell; elongate penial filament; and small size of the terminal gland on penis.

##### Description.

**Figure 9.**
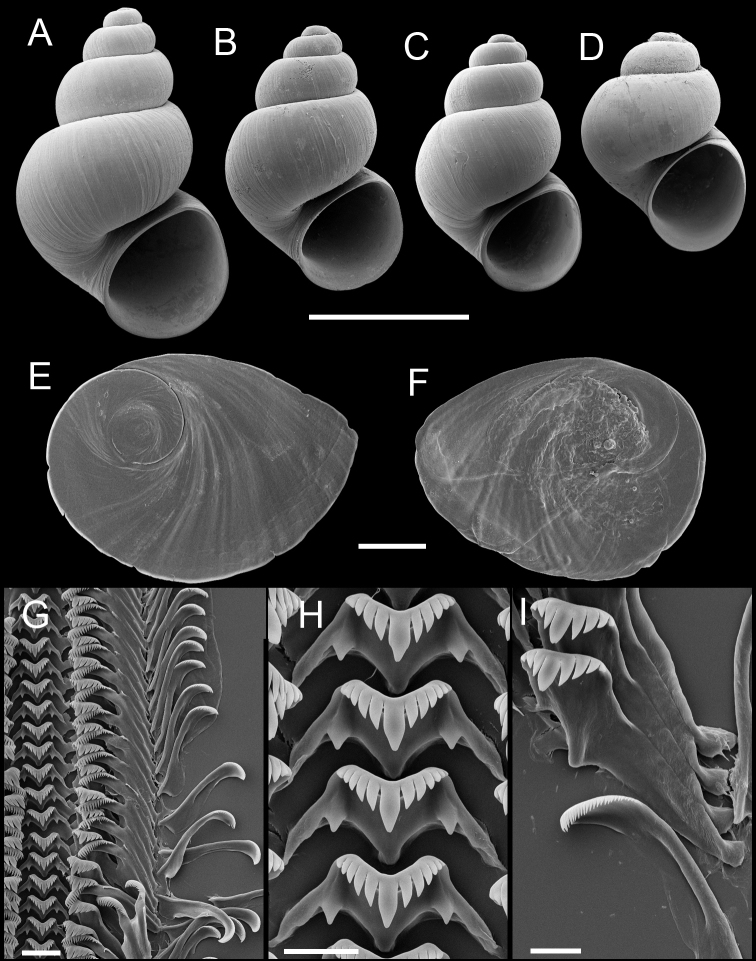
Shells, opercula and radula, *Pyrgulopsis turbatrix*. **A** Paratype, USNM 860699. **B–D** Shells, SBMNH uncat., USNM 853508, USNM 853510 **E, F** Opercula (outer, inner sides), USNM 860699 **G** Portion of radular ribbon, USNM 860699 **H** Central teeth, USNM 860699 **I** Lateral and outer marginal teeth, USNM 860699. Scale bars **A–D** 1.0 mm; **E, F** 250 µm; **G** 20 µm; **H, I** 10 µm.

Shell (Fig. 9A–D) ovate to narrow conic, rarely broadly conic, whorls 4.25–5.25. Teleoconch whorls strongly convex, shouldered. Aperture ovate, parietal lip complete, usually slightly disjunct, last 0.25 whorl sometimes loosened behind body whorl, umbilicus narrow. Outer lip thin, prosocline.

Operculum (Fig. 9E–F) as for genus; edges of last 0.5 whorl frilled on outer side; muscle attachment margins slightly thickened on inner side. Radula (Fig. 9G–I) as for genus; dorsal edge of central teeth moderately to highly concave, lateral cusps three–seven, basal cusps one–two. Lateral teeth having two–six cusps on inner sides and three–six cusps on outer sides. Inner marginal teeth with 14–31 cusps, outer marginal teeth with 15–33 cusps. Radula data are from USNM 857968, USNM 860450, USNM 860699, USNM 883373.

Penis (Fig. 4I–L) medium-sized; filament long, narrow, tapering, oblique; lobe medium-sized, tapering, slightly oblique; ventral surface of lobe having a small (terminal) gland (199/200 specimens) and rarely one or two additional glandular dots (3 specimens), dorsal surface sometimes having a small (penial) gland at base of filament (24/200 specimens) and rarely having an additional glandular dot (one specimen). Penial data are from USNM 854967, USNM 857969, USNM 860450, USNM 860699, USNM 883373, USNM 883981, USNM 1002785.

##### Distribution.

Spring Mountains region (Frenchman Flat; Indian Springs, Las Vegas, Pahrump Valleys [*Pyrgulopsis turbatrix*]), San Bernardino Mountains (Mojave, Southern California Coastal drainages [M19, M20, M21]), central Death Valley region (Amargosa River drainage, Panamint and Saline Valleys [M9-M22, M24, M27, M28]). The populations from the latter two areas were previously assigned to *Pyrgulopsis micrococcus*.

##### Remarks.

The penial gland was not observed in >25% of the males in any of the seven samples that we studied and consequently has been removed from the diagnosis. The three geographically separated subunits of *Pyrgulopsis turbatrix* are somewhat diverged genetically—e.g., mean sequence divergence is 0.9+/-0.2% (ranging from 1.1–1.5%) for COI and 0.9+/-0.2% (ranging from 1.1–1.3%) for NDI, but we have not found any consistent morphological differences among them.

## Discussion

The three novelties described herein increase the number of *Pyrgulopsis* species in the Death Valley region to 17 (Hershler and Sada 2002) and add to the large body of evidence supporting recognition of this desert area as one of the most significant hotspots of rarity and richness in the United States (Chaplin et al. 2000). Our revision of *Pyrgulopsis micrococcus* obviously is not yet complete as we have not treated clade F, which differs from the other lineages of *Pyrgulopsis micrococcus* (in the broad sense) by 4.3–12.6% for COI and 7.4–13.3% for NDI (Appendix II). The two populations in clade F differ by only 0.8+/-0.3% for COI and 1.2+/-0.5% for NDI, suggesting that they may be conspecific. Additional studies are needed to clarify the taxonomy of this clade and to evaluate the biogeographic cause of its broadly disjunct distribution.

All of the new species described herein are endemic to the Amargosa River basin. *Pyrgulopsis perforata* and *Pyrgulopsis licina* are distributed entirely within the confines of Death Valley National Park and Ash Meadows National Wildlife Refuge, respectively, and consequently are being afforded some measure of protection. The *Pyrgulopsis licina* populations are also being monitored by The Nature Conservancy as part of their Nevada Springs Conservation Plan (Abele 2011). Three of the five genetically differentiated lineages of *Pyrgulopsis sanchezi* are distributed in the land management areas mentioned above. The Tecopa lineage (M25) is distributed on private and public water resources lands and is being monitored as part of the Bureau of Land Management’s Amargosa River Area of Environmental Concern Implementation Plan (BLM 2006). The Shoshone lineage (M26) lives in a spring on private land that has a long history of disturbance which includes diversion of most of its flow and extensive modification of its outflow channel (USFWS 1984, Castleberry et al. 1990). This population appears to be restricted at present to a small area of leakage from a spring box (RH, HPL, CB, personal observation 15.XI.2011) and some measures will need to be taken to ensure its long term persistence. Our finding that *Pyrgulopsis micrococcus* is restricted to its type locality area (Oasis Valley) suggests a need for additional conservation-related activities. The known populations of this species are in disturbed springs (per Maciolek 1983) on private land and some protection is needed to ensure their long term persistence. Surveys are also needed to evaluate the current status of several populations of this species that have not been sampled for several decades (e.g., that in Ute Springs) and to determine whether there may be previously unrecorded populations in Oasis Valley, which contains a large area of groundwater discharge (Reiner et al. 2002). *Pyrgulopsis micrococcus* (as currently constituted) was a candidate for addition to the Federal List of Endangered and Threatened Wildlife and Plants (USFWS 1976) prior to its removal from this list in 1994 (USFWS 1994).

## Supplementary Material

XML Treatment for Pyrgulopsis licina

XML Treatment for Pyrgulopsis perforata

XML Treatment for Pyrgulopsis sanchezi

XML Treatment for Pyrgulopsis micrococcus

XML Treatment for Pyrgulopsis turbatrix

## Appendix I

Specimen codes, locality details and GenBank accession numbers for COI and NDI sequences. (doi: 10.3897/zookeys.330.5852.app1) File format: Microsoft Word file (doc).

## Appendix II

Genetic distances (maximum composite likelihood) of clades A–F (“*Pyrgulopsis micrococcus* complex”). (doi: 10.3897/zookeys.330.5852.app2) File format: Microsoft Word file (doc).
