# Supplementary material for: Systematics of a widely distributed western North American springsnail, Pyrgulopsis micrococcus (Caenogastropoda, Hydrobiidae), with descriptions of three new congeners
Source: Zookeys. 2013 Sep 10;(330):27–52. doi: 10.3897/zookeys.330.5852 (PMC3800804; doi:10.3897/zookeys.330.5852)
Supplement: Supplementary file 6 — Specimen codes, locality details and GenBank accession numbers for COI and NDI sequences. (doi: 10.3897/zookeys.330.5852.app1) File format: Microsoft Word file (doc). [file ZooKeys-330-027-s001.doc]

**Appendix 1**. Specimen codes, locality details and GenBank accession numbers for COI and NDI sequences.

| Code | Locality | USNM # | COI | NDI |
| --- | --- | --- | --- | --- |
| Clade A |  |  |  |  |
| M1 | Spring, Fleur de Lis Ranch, Oasis Valley, Nye Co., NV | 894330 | AY367415 (*n*=8, M1A-1D, G, K, N, O)  AY367418 (*n*=2, M1E-F)  AY367421 (*n*=6, M1H- J, L-M, P) | AY367492 (*n*=2, M1A-B) |
|  |  | 1004184 | AF520944 | — |
| M31 | Goss Springs, Oasis Valley, Nye Co., NV | 1004183 | AY367478 (M31A) | — |
|  |  |  |  |  |
| Clade B |  |  |  |  |
| M3 | Spring east of Scotty’s Castle, Grapevine Canyon, Death Valley, Inyo Co., CA | 894332 | AY367433 (*n*=2, M3A-B) | AY367498 (*n*=4, M3A-D) |
| M4 | Spring west of Scotty’s Castle, Grapevine Canyon, Death Valley, Inyo Co., CA | 894333 | AY367435 (M4A)  AY367436 (M4B) | AY367502 (*n*=2, M4A, D)  AY367503 (M4B)  AY367504 (M4C) |
| M5 | Surprise Spring, Death Valley, Inyo Co., CA | 894334 | AY367437 (M5A) | AY367506 (*n*=3, M5A-C) |
|  |  |  |  |  |
| Clade C |  |  |  |  |
| M7 | Last Chance Spring, Ash Meadows, Nye Co., NV | 894336 | AY367438 (M7A)  AY367439 (M7B) | — |
| M29 | Spring east of Crystal Reservoir, Ash Meadows, Nye Co., NV | 903982 | AY367476 (M29A) | — |
| M30 | Spring south of Clay Pits, Ash Meadows, Nye Co., NV | 903997 | AY367477 (M30A) | AY367536 (M30A) |
| M52 | Spring ca. 100 m north of Collins Ranch, Ash Meadows, Nye Co., NV | 1197780 | KF559189 (*n*=2, M52B-C) | KF559194 (*n*=2, M52B-C) |
| M54 | Spring south of Rogers Spring, Ash Meadows, Nye Co., NV | 1197782 | KF559190 (*n*=3, M54A-C) | KF559195 (*n*=2, M54A-C) |
| M58 | Spring east of Crystal Reservoir, Ash Meadows, Nye Co., NV | 1197775 | KF559191 (*n*=2, M58B-C) | KF559196 (*n*=2, M58B-C) |
|  |  |  |  |  |
| Clade D |  |  |  |  |
| M9 | Stream below Darwin Falls, Panamint Valley, Inyo Co., CA | 894338 | AY367441 (*n*=2, M9A-B) | — |
| M10 | China Garden Spring, Panamint Valley, Inyo Co., CA | 894339 | AY367443 (*n*=2, M10A-B) | AY367509 (M10A) |
| M11 | Spring above Darwin Falls, Panamint Valley, Inyo Co., CA | 894340 | AY367445 (M11A) | AY367510 (M11A) |
| M12 | Lower spring, Johnson Canyon, Death Valley, Inyo Co., CA | 894341 | AY367446 (*n*=2, M12A-B) | AY367511 (*n*=2, M12A-B) |
| M13 | Stream, Hanaupah Canyon, Death Valley, Inyo Co., CA | 894342 | AY367448 (*n*=4, M13A, C-E)  AY367449 (M13B)  AY367453 (M13F) | AY367513 (*n*=5, M13B-F) |
| M14 | Stream, Jail Canyon, Panamint Valley, Inyo Co., CA | 894343 | AY367454 (M14A)  AY367455 (M14B) | AY367518 (M14B) |
| M15 | Stream, Hall Canyon, Panamint Valley, Inyo Co., CA | 894334 | AY367456 (M15A)  AY367457 (M15B | AY367519 (M15A) |
| M16 | Spring, Snow Canyon, Panamint Valley, Inyo Co., CA | 894345 | AY367458 (M16A) | AY367520 (M16A) |
| M17 | Cottonwood Spring, Wildrose Canyon, Panamint Valley, Inyo Co., CA | 894346 | AY367459 (M17A) | AY367521 (M17A) |
| M18 | Saline Marsh, Saline Valley, Inyo Co., CA | 894347 | AY367460 (M18A) | AY367522 (M18A) |
| M19 | Cushenbury Springs, San Bernardino Mountains, San Bernardino Co., CA | 894348 | AY367461 (M19A) | AY367523 (M19A) |
| M20 | Springs at Big Bear Ranger Station, San Bernardino Mountains, San Bernardino Co., CA | 894349 | AY367462 (M20A) | AY367524 (M20A) |
| M21 | Springs, Mill Creek Canyon, San Bernardino Mountains, San Bernardino Co., CA | 894350 | AY367463 (*n*=2, M21A-B) | AY367525 (M21A) |
| M22 | Spring, Knight Canyon, Panamint Valley, Inyo Co., CA | 894351 | AY367465 (*n*=2, M22A-B)  AY367467 (M22C) | AY367526 (M22A) |
| M24 | Tennessee Spring, Panamint Valley, Inyo Co., CA | 894353 | AY367468 (M24A) | AY367527 (*n*=2, M24A-B) |
| M27 | Stream below Limekiln Spring, Panamint Valley, Inyo Co., CA | 894356 | AY367474 (M27A) | AY367534 (M27A) |
| M28 | Brewery Spring, Surprise Canyon, Panamint Valley, Inyo Co., CA | 894357 | AY367475 (M28A) | AY367535 (M28A) |
| *P. turbatrix* (P11) | Horseshutem Springs, Pahrump Valley, Nye Co., NV | 903989 | AF520936 (P11A) | AY367555 (P11A) |
| *P. turbatrix* (P84) | Cold Creek Spring, Indian Springs Valley, Clark Co., NV | 894822 | DQ364014 (P84A) | DQ364035 (P84A) |
| *P. turbatrix* (P86) | Spring, Lost Creek Canyon, Las Vegas Wash, Clark Co., NV | 894823 | DQ364015 (P86A) | DQ364036 (P86A) |
| *P. turbatrix* (P196) | Grapevine Springs, Amargosa River basin, Nye Co., NV | 1074291 | DQ364000 (P196D) | DQ364021 (P196D) |
|  |  |  |  |  |
| Clade E |  |  |  |  |
| M2 | Grapevine Springs, Death Valley, Inyo Co., CA | 894331 | AY367430 (*n*=15, M2A-O) | AY367494 (*n*=15, M2A-O) |
| M25 | Spring north of Tecopa Hot Springs, Amargosa River basin, Inyo Co., CA | 894354 | AY367469 (M25A)  AY367470 (*n*=8, M25B-C, AB-AD, AG-AI) | AY367529 (n=10, M25A, C, AA-AD, AF-AI) |
| M26 | Shoshone Spring, Amargosa River basin, Inyo Co., CA | 894355 | AY367472 (*n*=5, M26A, AD-AE, AG, AI)  AY367476 (*n*=2, M26B, AC)  KF559187 (*n*=5, M26AA-AB, AF, AH, AJ)  KF559188 (*n*=1, M26AK) | AY367531 (*n*=3, M26A-C)  KF559200 (*n*=11, M26AA-AK) |
| M51 | Spring ca. 100 m north of Collins Ranch, Ash Meadows, Nye Co., NV | 1204752 | KF559184 (*n*=2, M51A-B) | KF559197 (*n*=3, M51A-C) |
| M53 | Spring south of Rogers Spring, Ash Meadows, Nye Co., NV | 1204755 | KF559185 (*n*=3, M53A-C) | KF559198 (*n*=2, M53A-B) |
| M57 | Spring east of Crystal Reservoir, Ash Meadows, Nye Co., NV | 1197773 | KF559186 (*n*=2, M57A-B) | KF559199 (*n*=2, M57A-B) |
| M8 | Purgatory Spring, Ash Meadows, Nye Co., NV | 894337 | AY367440 (M8A)  DQ364001 (*n*=9, P197A, M8AA-AH) | DQ364022 (*n*=11, P197A, M8AA-AJ) |
| SS | Saratoga Spring, Death Valley, San Bernardino Co., CA | 1152506 | AY367480 (SS1)  DQ364003 (SS2) | AY367538 (SS1)  DQ364024 (SS2) |
|  |  |  |  |  |
| Clade F |  |  |  |  |
| M56 | Grapevine Springs, Death Valley, Inyo Co., CA | 1204756 | KF559192 (*n*=3, M56A-C) | KF559201 (*n*=3, M56A-C) |
| P29 | Spring tributary to Sisar Creek, Southern California coastal drainage, Ventura Co., CA | 905259 | GQ275093 | KF559202 |
|  |  |  |  |  |
| Outgroup taxa |  |  |  |  |
| *P. amargosae* | Saratoga Spring, Death Valley, San Bernardino Co., CA | 1008730 | AY367479 | AY367537 |
| *P.* “*amargosae*” (1) | Third spring along trail (from north), Amargosa Canyon, Inyo Co., CA | 1004042 | DQ364002 | DQ364023 |
| *P.* “*amargosae*” (2) | Spring, Amargosa Canyon, Inyo, Co., CA | 894693 | KF559193 | AY367539 |
| *P. anatina* | Spring southeast of Old Collins Spring, Railroad Valley, Nye Co., NV | 894713 | AY627926 | AY628048 |
| *P. arizonae* | Medicine Spring, Bylas, Gila River basin, Graham Co., AZ | 1003881 | AY627948 | AY628072 |
| *P. bacchus* | Tassi Spring, Grand Wash, Colorado River basin, Mojave Co., AZ | —* | DQ364005 | DQ364026 |
| *P. bifurcata* | Springs west of Carico Lake, Carico Lake basin, Lander Co., NV | 894715 | AY627925 | AY628047 |
| *P. californiensis* | Spring tributary to Campo Creek, Southern California coastal drainage, San Diego Co., CA | 899093 | AY627924 | AY628046 |
| *P. castaicensis* | Middle Canyon Spring, Southern California coastal drainage, LA Co., CA | 1123595 | GQ275097 | — |
| *P. conica* | Dripping Spring, Sacramento Wash, Mohave Co., AZ | —* | AY627958 | AY628083 |
| *P. crystalis* | Crystal Pool, Ash Meadows, Nye Co., NV | 903994 | AY367482 | AY367541 |
| *P. diablensis* | Stream, Del Puerto Canyon, San Joaquin River basin, Stanislaus Co., CA | 903985 | AY627922 | AY628044 |
| *P. dixensis* | Spring west of Cain Mountain, Dixie Valley, Pershing Co., NV | 1002470 | AY627946 | AY628070 |
| *P. erythropoma* | Kings Pool (outflow), Point of Rocks, Ash Meadows, Nye Co., NV | 903983 | AY367484 | AY367543 |
| *P. fairbanksensis* | Fairbanks Spring, Ash Meadows, Nye Co., NV | 903995 | AY627921 | AY628043 |
| *P. giulianii* | Stream, Sand Canyon, Indian Wells Valley (Northern Mojave basin), Kern Co., CA | 894352 | AF520937 | AY367545 |
| *P. glandulosa* | Nelson Place Spring, Verde River basin, Yavapai Co., AZ | —* | AY627959 | AY628084 |
| *P. intermedia* | Crooked Creek, Owyhee River drainage, Malheur Co., OR | 863510 | AY379442 | AY426385 |
| *P. isolatus* | Spring south of Clay Pits, Ash Meadows, Nye Co., NV | 903987 | AY367486 | AY367547 |
| *P. longinqua* | Spring west-southwest of Hunters Spring, Salton Sea basin, Riverside Co., CA | 903990 | DQ364006 | DQ364027 |
| *P. milleri* | Pierpoint Spring, Tulare-Buena Vista Lakes drainage, Tulare Co., CA | 905257 | GQ275096 | KF559203 |
| *P. montana* | Spring, upper Camp Valley, Meadow Valley Wash, Lincoln Co., NV | 894876 | AY627940 | AY628064 |
| *P. morrisoni* | Spring, Bubbling Pond Hatchery, Verde River basin, Yavapai Co., AZ | 1003880 | DQ364007 | DQ364028 |
| *P. nanus* | Five Springs, Ash Meadows, Nye Co., NV | 903993 | AY367487 | AY367548 |
| *P. owensensis* | Stream, canyon south of Piute Creek, Owens Valley, Mono Co., CA | 899099 | AF520922 | AY367549 |
| *P. pellita* | Sullivan Spring, Antelope Valley, Eureka Co., NV | 1002339 | AY627943 | AY628067 |
| *P. perturbata* | Northeast Spring, Fish Slough, Owens River basin, Mono Co., CA | 899098 | AY367488 | AY367550 |
| *P. pisteri* | Marsh Spring, Ash Meadows, Nye Co., NV | 1004041 | DQ364004 | DQ364025 |
| *P. robusta* | Polecat Creek, Snake River basin, Teton Co., WY | 905297 | AF520949 | AY426395 |
| *P. simplex* | Spring near Strawberry, Verde River basin, Gila Co., AZ | 1003862 | AY627949 | AY628073 |
| *P. sola* | Brown Springs, Verde River basin, Yavapai Co., AZ | —* | AY627957 | AY628082 |
| *P. sp.* | Grapevine Springs, Amargosa River basin, Nye Co., NV | 1074291 | DQ363999 | DQ364020 |
| *P. stearnsiana* (WC) | Springs, Wildcat Canyon, El Sobrante, San Francisco Bay drainage, Contra Costa Co., CA | 894694 | AF520925 | AY367551 |
| *P. stearnsiana* (PC) | Partington Creek, Central California coastal drainage, Monterey Co., CA | 905251 | AY367489 | AY367552 |
| *P. stearnsiana* (CC) | Stream, Colson Canyon, Central California coastal drainage, Santa Barbara Co., CA | 905256 | AY367490 | AY367553 |
| *P. taylori* | Spring tributary to San Luis Obispo Creek, Central California coastal drainage, San Luis Obispo Co., CA | 903986 | AY627923 | AY628045 |
| *Floridobia floridana* | Juniper Springs, St. Johns River basin, Marion Co., FL | 894755 | AF520916 | AY628035 |
| *Floridobia winkleyi* | Salt marsh, Scarborough, Saco River basin, Cumberland Co., ME | 883964 | AF520917 | AY628036 |

Sample sizes are given (in parentheses) after GenBank accession numbers when >1. The letter following the sample code identifies individual specimens.

“—“ indicates no data.

“—*” indicates DNA sample only.
